# Feedback Related Potentials for EEG-Based Typing Systems

**DOI:** 10.3389/fnhum.2021.788258

**Published:** 2022-01-25

**Authors:** Paula Gonzalez-Navarro, Basak Celik, Mohammad Moghadamfalahi, Murat Akcakaya, Melanie Fried-Oken, Deniz Erdoğmuş

**Affiliations:** ^1^Cognitive Systems Laboratory, Northeastern University, Boston, MA, United States; ^2^CAMBI (Consortium for Accessible Multimodal Brain-Body Interfaces), Portland, OR, United States; ^3^Electrical and Computer Engineering Department, University of Pittsburgh, Pittsburgh, PI, United States; ^4^Institute on Development and Disability, Oregon Health & Science University, Portland, OR, United States

**Keywords:** error related potentials, feedback related potentials, event related potentials, electroencephalography, brain computer interfaces, RSVP Keyboard^TM^, Bayesian fusion

## Abstract

Error related potentials (ErrP), which are elicited in the EEG in response to a perceived error, have been used for error correction and adaption in the event related potential (ERP)-based brain computer interfaces designed for typing. In these typing interfaces, ERP evidence is collected in response to a sequence of stimuli presented usually in the visual form and the intended user stimulus is probabilistically inferred (stimulus with highest probability) and presented to the user as the decision. If the inferred stimulus is incorrect, ErrP is expected to be elicited in the EEG. Early approaches to use ErrP in the design of typing interfaces attempt to make hard decisions on the perceived error such that the perceived error is corrected and either the sequence of stimuli are repeated to obtain further ERP evidence, or without further repetition the stimulus with the second highest probability is presented to the user as the decision of the system. Moreover, none of the existing approaches use a language model to increase the performance of typing. In this work, unlike the existing approaches, we study the potential benefits of fusing feedback related potentials (FRP), a form of ErrP, with ERP and context information (language model, LM) in a Bayesian fashion to detect the user intent. We present experimental results based on data from 12 healthy participants using RSVP Keyboard™ to complete a copy-phrase-task. Three paradigms are compared: [P1] uses only ERP/LM Bayesian fusion; [P2] each RSVP sequence is appended with the top candidate in the alphabet according to posterior after ERP evidence fusion; corresponding FRP is then incorporated; and [P3] the top candidate is shown as a prospect to generate FRP evidence only if its posterior exceeds a threshold. Analyses indicate that ERP/LM/FRP evidence fusion during decision making yields significant speed-accuracy benefits for the user.

## 1. Introduction

Event related potentials (ERPs) are commonly employed in the design of non-invasive electroencephalography (EEG)-based brain computer interfaces (BCIs) to detect the user intent (Farwell and Donchin, 1988; Acqualagna et al., 2010; Orhan et al., 2012; Akcakaya et al., 2014; Moghadamfalahi et al., 2015). The pioneer study from Donchin and Farewell demonstrated that ERPs can be used to design a letter by letter typing BCI (Farwell and Donchin, 1988). In addition to event related potentials (ERPs), depending on the BCI application, error-related potentials (ErrPs) can be used to indicate a perceived error. ErrPs are detectable as deflections in the EEG signal measured over the scalp of a person when they make or perceive an error (Falkenstein et al., 2000; Davies et al., 2004; Buttfield et al., 2006; Yazicioglu et al., 2006; Ferrez and del R. Millan, 2008; Gürel and Mehring, 2012; Margaux et al., 2012; Spüler et al., 2012; Kieffaber et al., 2016). Different variants of ErrPs can be measured in recorded EEG signal. For example, when the user realizes that the interface failed to properly recognize user's intention, an ErrP signal is induced, which can characterized by two fronto-central positive peaks appearing 200 and 320 ms after the feedback; a fronto-central negativity near 250 ms and at last, broader fronto-central negative deflection about 450 ms after the feedback. These latencies can change depending on the experimental paradigm (Iturrate et al., 2013). Moreover, some studies have demonstrated correlation between trial-by-trial estimates of the ErrP and the post-error slowing (Debener et al., 2005). Based on these studies, it has been proposed that the negative deflection of the ErrP signal is the result of an error-detection mechanism, as opposed to being an inhibitory or corrective signal. In addition, it has been studied that the positive components of the ErrP reflects conscious error processing or post-error adjustment of response strategies (Falkenstein et al., 2000).

While some BCI typing systems have shown encouraging results (Kawala-Sterniuk et al., 2021), there is still much work to be done to produce real-world-worthy systems that can be comfortably, conveniently, and reliably used by individuals with severe neuromuscular disabilities who cannot use standard communication pathways or other assistive technologies. This work presents several improvements to a language-model-assisted EEG-based typing BCI, RSVP Keyboard™ (Moghadamfalahi et al., 2015), as well as similar designs that depend on visually evoked P300 potentials. The baseline system fuses text/language and EEG evidence to infer user intent in EEG-controlled spelling to generate expressive language. In particular, we study the potential benefits of fusing feedback related potentials, a form of ErrP, with ERP and context information (language model, LM) in a Bayesian framework. The probabilistic evidence for ERP, ErrP, and non-EEG are computed using different probabilistic generative models.

We represent the domain knowledge and casual relationship among difference variables in a probabilistic graphical model. The presented approach is a general dynamic fusion framework that could be used with various presentation paradigms. Typing interfaces aim to reach a certain confidence level before making a decision on the user intent, and accordingly, sequences of symbols are repeated multiple times. In our approach, after every presented sequence, we compute the posterior distribution of the symbol set (all the symbols in the English alphabet and the backspace symbol) conditioned on ERP likelihoods and LM-based priors. The mode of posterior distribution is selected as prospect symbol that is presented to the user, either after every sequence or after a confidence threshold is reached. The prospect symbol is an additional visual stimuli, which induces an EEG response that is indicative of that prospect's correctness. We refer to this response as feedback related potential (FRP), which takes the form of an ErrP/non ErrP indicating an incorrect/correct prospect symbol being presented. After the prospect symbol is presented and the new FRP evidence is obtained, through the Bayesian graphical model, the FRP evidence is fused with the EEG and LM-based evidence and the posterior distribution of the symbols is updated. Given the low signal-to-noise-ratio of EEG, we take an iterative update approach by presenting multiple sequences of ERP and FRP stimuli to the user to compute a more robust estimate, until the posterior reaches an information theoretic confidence threshold. User intent is then selected using maximum a posteriori (MAP) inference.

Existing typing BCIs that attempt to use ERP/FRP jointly typically fall into one of these categories: a flag produced by the ErrP classifier results in (a) the deletion of the last selection made using the ERP classifier (Dal Seno et al., 2010; Schmidt et al., 2012; Spüler et al., 2012; Chavarriaga et al., 2014); (b) replacing the last selection made using the ERP classifier with the second probable option (Combaz et al., 2012; Margaux et al., 2012; Chavarriaga et al., 2014); (c) presenting more stimuli to gather additional ERP evidence, but not using the FRP to update symbol probabilities over the alphabet (Combaz et al., 2012). A language model is not fused with ERP evidence in these particular examples, but it has been suggested for boosting both ERP and FRP evidence assessment. Unlike these early attempts on using FRP evidence to make hard decisions based on ErrP classifier outputs, we seek Bayesian fusion of ERP, FRP, and language evidence using probabilistic generative models. The system presented in this paper automatically decides to select a letter to type or proceed with more ERP/FRP evidence collection in a probabilistic fashion.

In an earlier study, we observed the potential enhancements that can be achieved through a joint probabilistic inference from all evidences (i.e., FRP, ERP, and LM), rather than using FRP as a switch Gonzalez-Navarro et al. (2016a); Orhan et al. (2016). In the early study, Monte Carlo simulations are performed using synthetic EEG features from models calibrated with real ERP/FRP data, and the results are simulated for five users with synthetic EEG features (Gonzalez-Navarro et al., 2016a). As our simulation results suggested, Bayesian fusion of all evidence (FRP, ERP, and LM) yields faster typing speeds for all participants without compromising accuracy. On the other hand, use of ErrP in a sub-optimal fashion, by allowing FRP decisions to override ERP, also improved speed relative to not using FRP at all. But our results indicated that Bayesian fusion of FRP with ERP, and not treating the former as a de facto superior form of evidence, may yield better outcomes. Based on these results, we decided to conduct a new study, presented in this manuscript, to evaluate the performance of two different system strategies for a joint probabilistic inference framework. This is the first work where we study experimental results based on data from healthy participants. We study the potential benefits of fusing feedback related potentials (FRP) with ERP and context information (LM) in a Bayesian fashion to detect the user intent.

To illustrate the efficacy of our approach we use RSVP Keyboard™ (Moghadamfalahi et al., 2015), an EEG based BCI for letter by letter typing, which is described in more details in section 3. Three strategies [P1], [P2], and [P3] are compared in terms of speed, accuracy, and information transfer rate (ITR). The EEG for this study is acquired from 12 healthy participants using RSVP Keyboard™ to complete a copy-phrase-task. [P1], the baseline system fuses LM and ERP (collected from RSVPs) evidence in a Bayesian fashion to infer user intent. On the other hand, our novel propositions, [P2] and [P3], use a joint inference from all evidence (FRP, ERP, and LM) to make a decision. In [P2], FRP evidence is collected after every RSVP sequence; whereas in [P3], RSVP sequences are repeated multiple times until a confidence level is achieved, then the feedback is presented as the mode of estimated posterior (in other words, FRP evidence is collected less frequently in [P3]).

## 2. Proposed Graphical Model for Inference

### 2.1. Decision Framework

In a typical letter by letter typing BCI application, the user has to select among a discrete set of *task symbols* from a Dictionary D={A,B,…Z}∪{<,-} where “−” represents space symbol and “<” represents backspace symbol. Here, we examine how a BCI can infer a *task symbol* from different EEG evidence and prior context information. In particular, we build a decision framework that takes into account two types of EEG evidence: FRP and ERP evidence. We propose several methods for combining FRP, ERP evidence and prior context information, using real-time posterior probability updates. This BCI application utilizes a visual presentation module to detect the user intent and the EEG collected during the visual stimulation is then employed in decision making procedure.

Different visual presentation methods can be considered in order to evoke visual potentials. Rapid serial visual presentation (RSVP) paradigm is a minimally gaze dependent alternative for matrix presentation paradigms, that is aimed to induce ERPs for intent detection. In the RSVP paradigm, the symbols are rapidly presented as a time series on a prefixed location on the screen in a pseudo-random order, to evoke the response when the target symbol appears (Acqualagna et al., 2010; Orhan et al., 2012; Moghadamfalahi et al., 2015). In this presentation scheme, each flashing letter is a trial and in each “sequence,” a subset of dictionary is presented. From now on, we will be referring to only inducing ERP (target) evidence when we mention RSVP trial.

**Figure 1:**
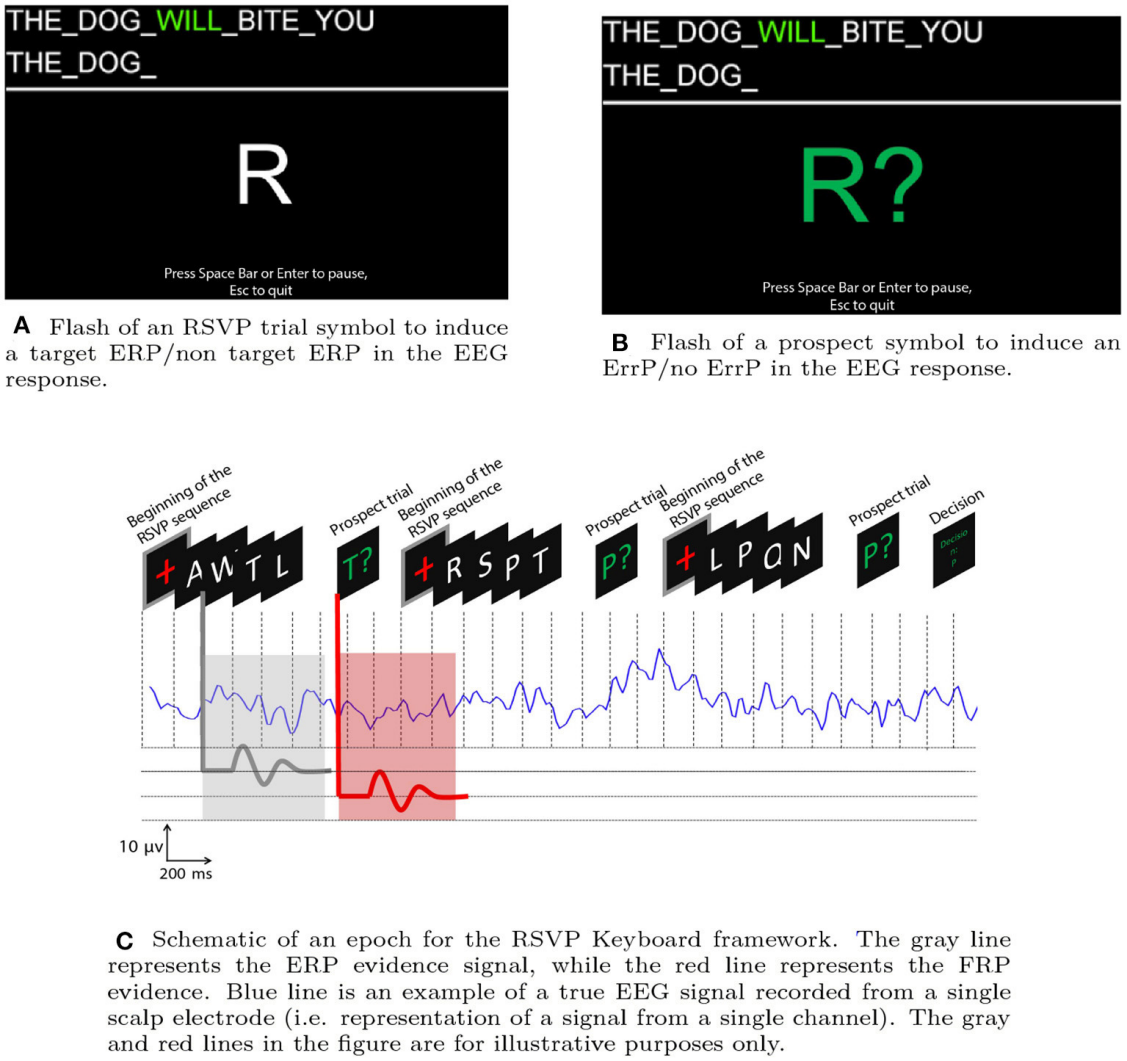
Different visual stimuli: **(A)** RSVP and **(B)** Feedback trials. **(C)** Schematic of an epoch with RSVP and Feedback sequences. A series of RSVP sequences including non-target and target symbols are shown at a prefixed position on the screen consecutively over time in rapid serial fashion. The RSVP sequence starts with a + symbol. At the end of each RSVP sequence, a prospect symbol is appended.

Figures 1A,B illustrate a flash of a prospect symbol and RSVP trial respectively. Due to low signal-to-noise-ratio (SNR) of EEG, the system usually requires to query the user with more than one “sequence” and “prospect symbol” to achieve a desired confidence level before making a decision. The set of “sequence” and “prospect symbol” which leads to a decision is called an “epoch.” In every epoch, it is assumed that the target symbol remains unchanged. Figure 1C represents a schematic of an EEG epoch in the RSVP Keyboard™ including a series of letters in an ERP sequence and a feedback stimulus as a “prospect symbol.” The feedback stimulus is always presented at the end of the RSVP sequence (shown in green). In Figures 1A,B, “Press Space Bar or Enter to pause” indicates the Pause/Play button. “Esc to quit” indicates the exit button should the participant choose to end the experimental session. Both options are added to the experimental design for the convenience of the user.

### 2.2. Probabilistic Graphical Model (PGM)

**Figure 2:**
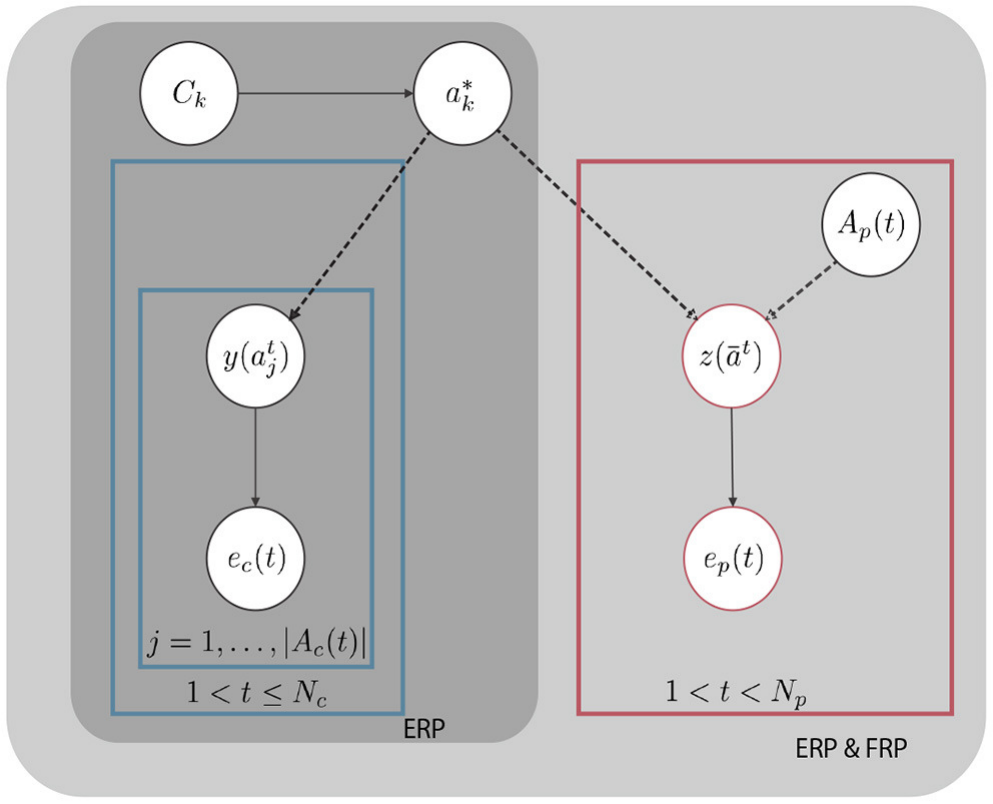
Proposed probabilistic graphical model representing the *k*th epoch. Here, the dashed lines show a deterministic relation while the solid lines define a probabilistic correspondence. *z* (ā^*t*^) = 1 ErrP label, *z* (ā^*t*^) = 0 non ErrP label. $y\;\left(a_{j}^{t}\right)=1$ target label, $y\;\left(a_{j}^{t}\right)=0$ non Target label. *t* denotes sequence index. *j* denotes trial index.

The proposed probabilistic graphical model (PGM) that represents *k*th “epoch” for an EEG-based typing application is presented in Figure 2.

Here, $a_{k}^{*}$ is a random variable which represents the user intent in epoch *k*, $A_{c}\left(t\right)=\left\{a_{j}^{t}|j=1,\ldots |A_{c}\left(t\right)|\right\}$ is a subset from the dictionary D, treated as the “sequence” at instant *t* of the epoch *k*, c denotes for candidate, |*A*_*c*_(*t*)| is the number of symbols presented in the *t*-th sequence, $C_{k}$ represents the context information that has been provided with the language model for which we will provide a brief description, in section 2.5. Moreover, here we introduce $A_{p}\left(t\right)\in D$. This set is a singleton $A_{p}\left(t\right)=\left\{{ā}^{t}\right\}$ which includes the prospective symbol for the query set *A*_*p*_(*t*) at instant *t* (p denotes for prospect). In addition, $e_{c}\left(a_{j}^{t}\right)$ and $e_{p}\left({ā}^{t}\right)$ are the ERP and FRP evidence obtained in response to an RSVP trial $a_{j}^{t}$ and feedback trial ā^*t*^ respectively. We assume that the user intent is not changing within an epoch. Hence, given that every $a_{j}^{t}\in A_{c}\left(t\right)$ and ${ā}^{t}\in A_{p}\left(t\right)$ are either target or non-target, the intent inference can be formulated as a binary decision problem. Therefore *y*(·), *z*(·) correspond to binary class labels for ERP, FRP responses. Hence, $y\left(a_{j}^{t}\right):=\delta \left(a_{j}^{t};a^{*}\right)$ has a one-to-one relationship with the true state $a_{k}^{*}$ such that $y\left(a_{j}^{t}\right)=1$ if $a_{k}^{*}=a_{j}^{t}$ and 0 otherwise. Similarly, *z*(ā^*t*^): = δ(ā^*t*^; a^*^). *N*_*c*_ and *N*_*p*_ are the maximum number of “sequences” and “prospect symbols” that can be used in an epoch if a desired confidence level is not reached in reasonable duration. In the case that we do not use FRP evidence, the right box from the graphical model from Figure 2 will be eliminated and the rest will remain the same. We utilize the graphical model presented to compute the posterior distribution of the intended character $a_{k}^{*}$ after collecting the EEG evidence and by utilizing the language model evidence. The details of the posterior distribution computation is given in section 2.4. In order to make inference on the user intent, we compare three different evidence acquisition paradigms (one for each strategy). These paradigms are discussed in section 2.3.

### 2.3. Evidence Acquisition Paradigms

**Figure 3:**
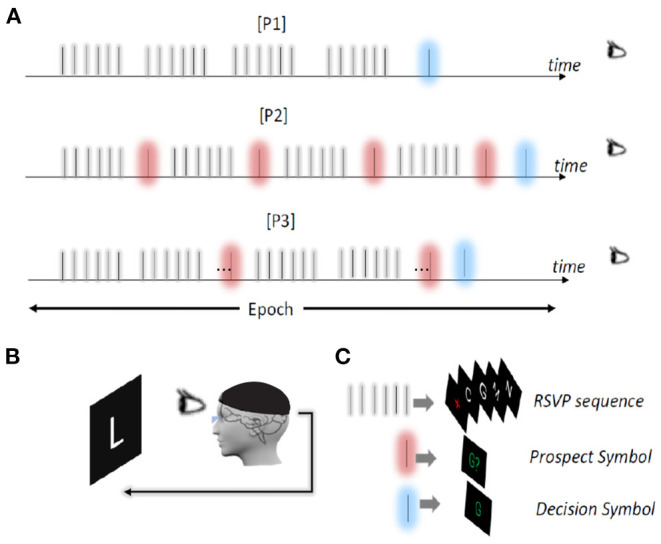
Evidence acquisition paradigms, experimental setup, and visual stimuli type. **(A)** Three evidence acquisition paradigms. First row shows [P1], second row shows [P2], and third row shows [P3]. **(B)** Visual stimuli are projected on a black screen. The EEG evidence collected after the presentation of a typical sequence and prospect symbol stimuli are used for detecting user intent. **(C)** Three different visual stimuli, RSVP sequence, prospect symbol, and decision symbol.

Here, we present three different evidence acquisition paradigms: (i) [P1], (ii) [P2], and (iii) [P3] as shown in Figure 3.

1. [P1] (*Baseline*): In this paradigm a set of pseudo-randomly ordered stimuli are presented to the user to elicit ERP. Each stimulus is a trial. Sets of trials that are presented with no time gaps are called a sequence *A*_*c*_(*t*). Every sequence can only contain up to one target stimulus. After each sequence, the posterior distribution over the character set is computed and a decision is made if the maximum probability exceeds a predefined threshold or a time limit is reached. Otherwise, the system continues with more sequences.
  This paradigm, is the baseline for RSVP Keyboard™ and it does not include FRP evaluation.
2. [P2] (*Always FRP*): In this paradigm we first query the user with ERP sequences in a similar fashion as [P1], then the mode of posterior is depicted as a *prospect symbol* i.e. *A*_*p*_(*t*). *A*_*p*_(*t*) is then presented on a prefixed location of the screen, like in regular RSVP trials, to induce FRP in EEG. Depending on the instructions given to the user, this FRP may take the form of an error-related potential (ErrP) indicating an incorrect prospect symbol being presented. The collected EEG in response to each prospect symbol is used to update the posterior using the PGM shown in Figure 2.
  This paradigm is also utilizes MAP inference, in a procedure similar to [P1].
3. [P3] (*Confirm FRP*): This paradigm is similar to [P1] and [P2] but the top candidate is shown as a *prospect symbol* to generate FRP evidence only if its posterior probability exceeds a threshold. The graphical model presented in Figure 2 is directly used to fuse the ERP and FRP evidence to infer the user intent.

### 2.4. Maximum a Posteriori (MAP) Inference

**Figure 4:**
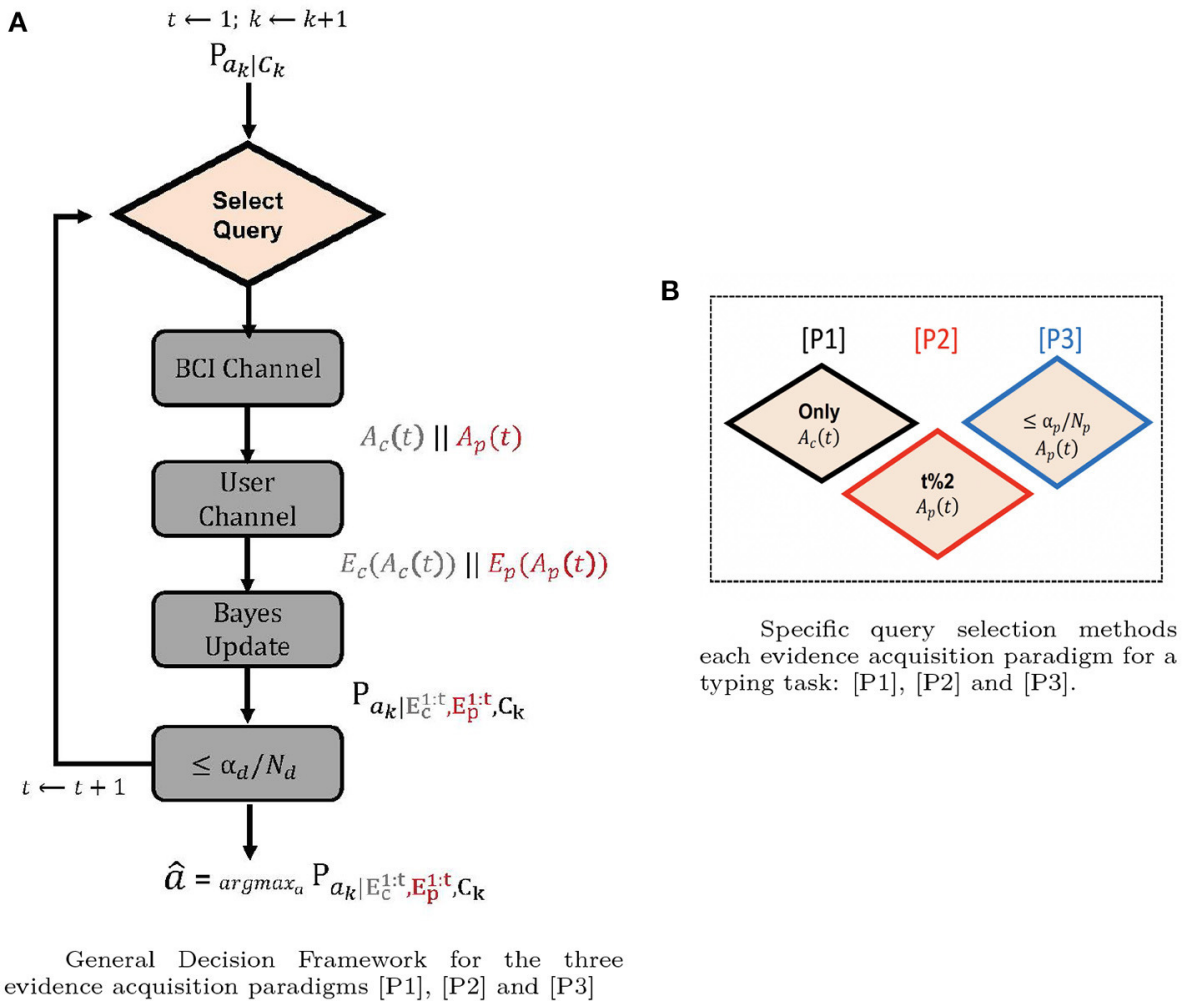
**(A)** General decision framework for the three evidence acquisition paradigms [P1], [P2], and [P3]. The only part that differs in each paradigm is the select query block. BCI channel decides which query is going to be presented, the evidence from the query is collected in the user channel, α_*d*_ is the decision threshold. *N*_*d*_ is the total number of sequences (including ERP + FRP). Decision is made when the posterior probability of the selected symbol passes the threshold α_*d*_, or when the total number of sequences is reached (denoted with ≤ α_*d*_/*N*_*d*_). In **(B)**, t%2 stands for t *mod* 2 (modulo operation), indicating that the prospect symbol is shown once after every RSVP sequence.

The decision making process utilizes a maximum a posteriori (MAP) inference mechanism for intent detection. The graphical model presented in Figure 2 is used to compute the posterior distribution of the intended symbol, after evaluating the ERP and FRP likelihoods in recorded EEGs during ERP and FRP sequences and using context priors. A general decision framework for the three evidence acquisition paradigms is presented in Figure 4. According to this framework, before making a final decision the ERP and FRP evidences corresponding to multiple sequences are aggregated and fused with the context prior. Different query selection methods [P_*i*_] *i* = {1, 2, 3} are presented in Figure 4. (Please see section 2.3 for more details.)

We estimate the prospective symbol ${ā}^{t}\in A_{p}\left(t\right)$ at instant *t*, as the mode of posterior distribution:


(1) $$\begin{array}{l} {ā}^{t}=\arg\underset{\text{a}\in D}{\max}P\left(a_{k}^{*}=\text{a}|E_{c}^{1:t},E_{p}^{1:t-1};C\right) \end{array}$$

where ${â}_{k}^{*}$ is the estimated user intent; $E_{c}^{1:t}={\left\{E_{c}\left(A_{c}\left(j\right)\right)\right\}}_{j=1}^{t}$ is the ERP evaluations for all the sequences in epoch *k* up to *t*; $E_{c}\left(A_{c}\left(t\right)\right)={\left\{e_{c}\left(a_{j}^{t}\right)\right\}}_{j=1}^{\left|A_{c}\left(t\right)\right|}$ is the set of observation for the query set *A*_*c*_(*t*); $E_{p}^{1:t-1}={\left\{E_{p}\left(A_{p}\left(j\right)\right)\right\}}_{j=1}^{t-1}$ is the FRP EEG evidences for all the observed prospective sequences in epoch *k* at instant *t*; $E_{p}\left(A_{p}\left(t\right)\right)=e_{p}\left({ā}^{t}\right)$ is the set of observation vectors for the prospective set *A*_*p*_(*t*). For [P2] and [P3] the FRP EEG evidence $e_{p}\left({ā}^{t}\right)$ is obtained in response to ā^*t*^.

To compute the posterior distribution in (1), we utilize the assumptions of the graphical model presented in Figure 2. According to this PGM, the ERP and FRP evidence and context information are independent when the intended symbol *a*_*k*_ is given. Then for epoch *k* and at time instant *t*, after observing the query sets *A*_*c*_(*t*) and *A*_*p*_(*t*), the maximum a posteriori can be computed using the objective function in (2).


(2) $$\begin{array}{l} {â}_{k}^{*}=\arg\underset{\text{a}\in D}{\max}P\left(E_{c}^{t}|a_{k}^{*}=\text{a}\right)\cdot P\left(E_{p}^{t}|a_{k}^{*}=\text{a}\right)\cdot P\left(a_{k}^{*}=\text{a}|C\right) \end{array}$$

We can further assume that conditioned on the unknown symbol *a*_*k*_ all EEG evidence from different trials are independent, and simplify the first two terms of Equation (2) as:


(3) $$\begin{array}{l} P\left(E_{c}^{t}|a_{k}^{*}=\text{a}\right)=\left(\underset{\begin{array}{c} t=1,\;\ldots ,N\\ \left\{j\;|a_{j}^{t}=\text{a},y\left(a_{j}^{t}\right)=1\right\} \end{array}}{\prod }\frac{p\;\left(e_{c}\left(a_{j}^{t}\right)|y\;\left(a_{j}^{t}\right)\right)}{p\;\left(e_{c}\left(a_{j}^{t}\right)|0\right)}\right) \end{array}$$

(4) $$\begin{array}{l} P\left(E_{p}^{t}|a_{k}^{*}=\text{a}\right)=\left(\underset{\begin{array}{c} t=1,\;\ldots ,N\\ \left\{{ā}^{t}=\text{a},z\left({ā}^{t}\right)=0\right\} \end{array}}{\prod }\frac{p\;\left(e_{p}\left({ā}^{t}\right)|z\;\left({ā}^{t}\right)\right)}{p\;\left(e_{p}\left({ā}^{t}\right)|1\right)}\right) \end{array}$$

According to the inference equation defined in (2), we need to estimate (i) the context prior that we estimated using a language model $P\left(a_{k}^{*}=\text{a}|C\right)$, (ii) class conditional distributions over the ERP evidence *p*(*e*_*c*_|1) for target and *p*(*e*_*c*_|0) for non-target classes, and (iii) class conditional distributions over the FRP EEG evidence *p*(*e*_*p*_|0) and *p*(*e*_*p*_|1). We have implemented the proposed ERP and FRP data acquisition paradigms using the RSVP Keyboard™ framework (Moghadamfalahi et al., 2015).

### 2.5. Context Information

To compute $P\left(a_{k}^{*}=\text{a}|C\right)$, we utilize an n-gram language model which provides a prior probability over every symbol in the dictionary. We have shown that context information when fused with EEG evidence improves the system performance effectively (Orhan et al., 2013; Moghadamfalahi et al., 2015). An n-gram LM is a Markov model of order *n* − 1. Let $C={\left\{a_{m}^{*}\right\}}_{m=n-1,\;\ldots ,\;1}$, where $a_{m}^{*}$ is the *m*^th^ previously typed character. Then:


(5) $$\begin{array}{l} P\left(a|C\right)=P\left(a|{\left\{a_{m}^{*}\right\}}_{m=n-1,\;\ldots ,\;1}\right)=\frac{P\left(a,a_{n-1}^{*},\;\ldots ,a_{1}^{*}\right)}{P\left(a_{n-1}^{*},\;\ldots ,a_{1}^{*}\right)} \end{array}$$

In our system, we use a 6-gram language model, which is trained on the NY Times portion of the English Gigaword corpus (Roark et al., 2010).

## 3. Human-in-the-Loop Experiments

We perform a set of online experiments to compare the effects of [P1], [P2], and [P3] on system performance. We collected data from 12 healthy participants (5 females), 22–38 years old. After a calibration session, participants were asked to perform a copy phrase task of RSVP Keyboard™. The data were collected according to the guidelines of an IRB-approved protocol at Northeastern University (IRB 130107).

### 3.1. Method

In RSVP Keyboard™, the EEG signal is acquired using a g.USBamp biosignal amplifier with active g.Butterfly electrodes at a sampling rate of 256 Hz, from 16 EEG sites (according to the International 10/20 configuration): Fp1, Fp2, F3, F4, Fz, Fc1, Fc2, Cz, P1, P2, C1, C2, Cp3, Cp4, P5, and P6. To improve the signal-to-noise ratio (SNR), and to eliminate drifts, signal is filtered by an FIR linear-phase bandpass filter with cutoff frequencies [1.5,42] Hz and a notch filter at 60 Hz. Typically, a wideband filter, such as a [0.05–30] Hz filter is recommended to avoid the potential distortion of ERP waveforms (Luck, 2014). In our work, temporal-windowed EEG signals are filtered by [1.5,42] Hz bandpass filter (FIR, linear phase, length 153, 0 DC-gain) to eliminate the low frequency deviations and high frequency noise. Lower high-cutoff frequencies may be used (Orhan et al., 2016).

In order to capture the ERP and FRP, while omitting the possible motor reposes (Moghadamfalahi et al., 2015), EEG from a time window of [0, 500) ms after each flash's onset is processed as the corresponding raw data for each trial. To further pre-process after filtering, the EEG data for each channel are first down-sampled by 2 and projected to a lower dimensional space using principal component analysis (PCA), and finally data from every channel is concatenated to form the feature vector ${\text{y}}_{j}^{i}$ for trial *i*th, of type *j* in response to a trial, as we defined in Equation (7). More specifically, ${\text{y}}_{p}^{i}$ represents FRP evidence for the prospective symbol trial *i*th; and ${\text{y}}_{c}^{i}$ represents ERP evidence for the query trial *i*th. After pre-processing,


(6) $$\begin{array}{l} {\text{y}}_{j}^{i}={\left[\begin{array}{c} {\text{v}}_{j}^{i}{\left[1\right]}^{T}\;{\text{v}}_{j}^{i}{\left[2\right]}^{T}\;\ldots \;{\text{v}}_{j}^{i}{\left[N_{ch}\right]}^{T} \end{array}\right]}^{T}\in {\mathbb{R}}^{N_{ch}\cdot N_{t}} \end{array}$$

where ${\text{v}}_{j}^{i}\left[n\right]$ is the multivariate measurement collected from channel *n*. Note that here *N*_*ch*_ = 16 is the number of channels and *N*_*t*_ is the number of time samples for each channel after applying PCA.

We then perform a quadratic projection of these feature vectors on to a one dimensional space so that it maximizes the separation between two possible classes of *non-target* and *target*. This projection is obtained as the log-likelihood ratio of two multivariate normal density functions estimated using regularized discriminant analysis (RDA) over target and non-target classes. $e_{j}^{i}\left(a_{c}^{i}\right)$ and $e_{j}^{i}\left(a_{p}^{i}\right)$, are the one dimensional ERP and FRP evidences, respectively. We estimate the class conditional distributions of *p*(*e*_*c*_(*a*)|1), *p*(*e*_*c*_(*a*)|0) over the ERP evidences; and *p*(*e*_*p*_(*a*)|1), *p*(*e*_*p*_(*a*)|0) over the FRP evidences, using kernel density estimation (KDE). We employ Gaussian kernel with a bandwidth computed using the Silverman's rule from the recorded labeled data (Silverman, 1986). Note that these distributions are computed after collecting data in a calibration session. Then, the estimated densities are used in test sessions.

Recall that the EEG (ERP and FRP) evidence and language model prior are fused using the assumptions of the graphical model presented in Figure 2 to obtain the posterior probability mass function (PMF). The posterior probabilities is then used in MAP inference framework to make a joint decision as described in section 2.

### 3.2. Experiment Design

All users participate in three *copy phrase* tasks, each task being performed on a separate day. In each day, the user performs the task pursuing one of [P1], [P2], and [P3] paradigms. The order of the paradigms are randomly assigned to the users to avoid the learning impact on the typing performance.

A *copy phrase* task includes typing the following ten different phrases.

**Figure 5:**
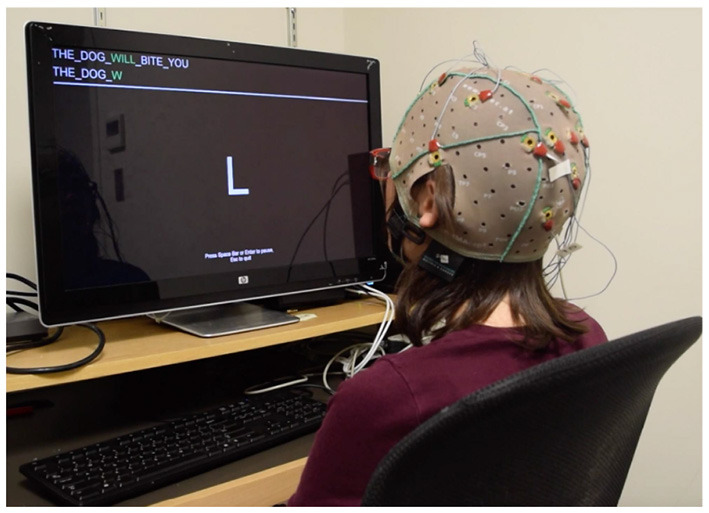
Copy phrase task performed on EEG-based BCI using RSVP Keyboard™ paradigm. The user is asked to type **WILL**.

1. THE DOG “**WILL**” BITE YOU,
2. GO TO “**THE**” MOVIES,
3. GOOD HEALTH “**CARE**” IS CRUCIAL,
4. SUPER “**BOWL**” SUNDAY,
5. EAT THREE TIMES A “**DAY**,”
6. THE THIRD “**SEAT**” FROM THE LEFT,
7. MY PARENTS “**FIND**” ME FUNNY,
8. SHE ALSO “**PAID**” FOR LUNCH,
9. SOMETHING THAT “**BUYS**” US TIME,
10. THE COMPOSER “**SITS**” QUIETLY,

Each phrase includes a missing word and the users are asked to complete these words. Here, the target words are written in bold. The entire sentence is shown to the user before each phrase is being typed. We use different phrases with different difficulty levels in terms of prior probability provided by the language model. For instance, the words such as “**THE**” or “**WILL**” are very easy to type because their initial letters are very likely based on the LM prior. However, the words such as “**PAID**” or “**BUYS**” are very difficult to type. Figure 5 demonstrates an example of a user performing the copy phrase task.

Prior to each copy phrase task all participants perform two *calibration* tasks: *calibration*_*ERP*_ and *calibration*_*FRP*_. *Calibration*_*ERP*_ is used to learn the statistics of the ERP classifier (target vs. non target), using the calibration mode of the system to record labeled EEG data. Typically, each *calibration*_*ERP*_ session consists of 100 sequences of symbols. Before each sequence, the user is asked to attend to a particular symbol. Then a sequence consisting of the target symbol and 9 other non-target symbols is presented to the user in a random order. *Calibration*_*FRP*_ is used to learn the statistics of the FRP classifier (correct vs. non correct) using the copy mode of the system to record labeled EEG data. To obtain compatible evidence, we simulated [P2] and [P3] paradigms to collect supervised FRP EEG data.

During *calibration*_*FRP*_, we modify the LM probabilities, in order to record enough labeled data for correct and non correct classes. Users are asked to rest between *calibrations* and *copy phrase* tasks and continue once they felt ready.

The length of each trial is 500 ms for all paradigms, there are 10 trials in one ERP sequence and 1 trial (i.e., the prospect symbol followed by a question mark) in one FRP sequence. *A*_*c*_(*t*) is selected based on the posterior probability (fusion of evidence + LM). In [P1], the trial symbol is shown for 150 ms followed by a 50 ms blank screen (i.e., the inter-trial interval). The interval between successive sequences is 500 ms. In [P2] and [P3], after ERP evidence is collected, the trial symbol is shown for 0.9 s followed by a 0.1s blank screen for the FRP evidence. Decision symbol is shown for 2 s. The maximum number of sequences allowed in an epoch is 100 for calibration tasks (Simply because during calibration, we have a single combined epoch and we do not make decisions). In copy phrase tasks, the maximum number of sequences allowed in an epoch is 8 (that is, a decision is made after max 8 sequences in an epoch). In paradigm [P3], the posterior probability for showing the prospect symbol is set as α_*p*_ = 0.66. Note that, we do not employ an α_*p*_ during paradigm [P2] because we already show the prospect symbol after every ERP sequence. For all three paradigms ([P1], [P2], and [P3]), the posterior probability threshold for decision is set as α_*d*_ = 0.9.

## 4. Analysis Results

Using the data collected in the human-in-the-loop copy phrase and calibration experiments described in section 3, we report the effect of the three evidence acquisition paradigms: [P1] (*Baseline*), [P2], and [P3].

### 4.1. Human-in-the-Loop Calibration Experiment Results

**Figure 6:**
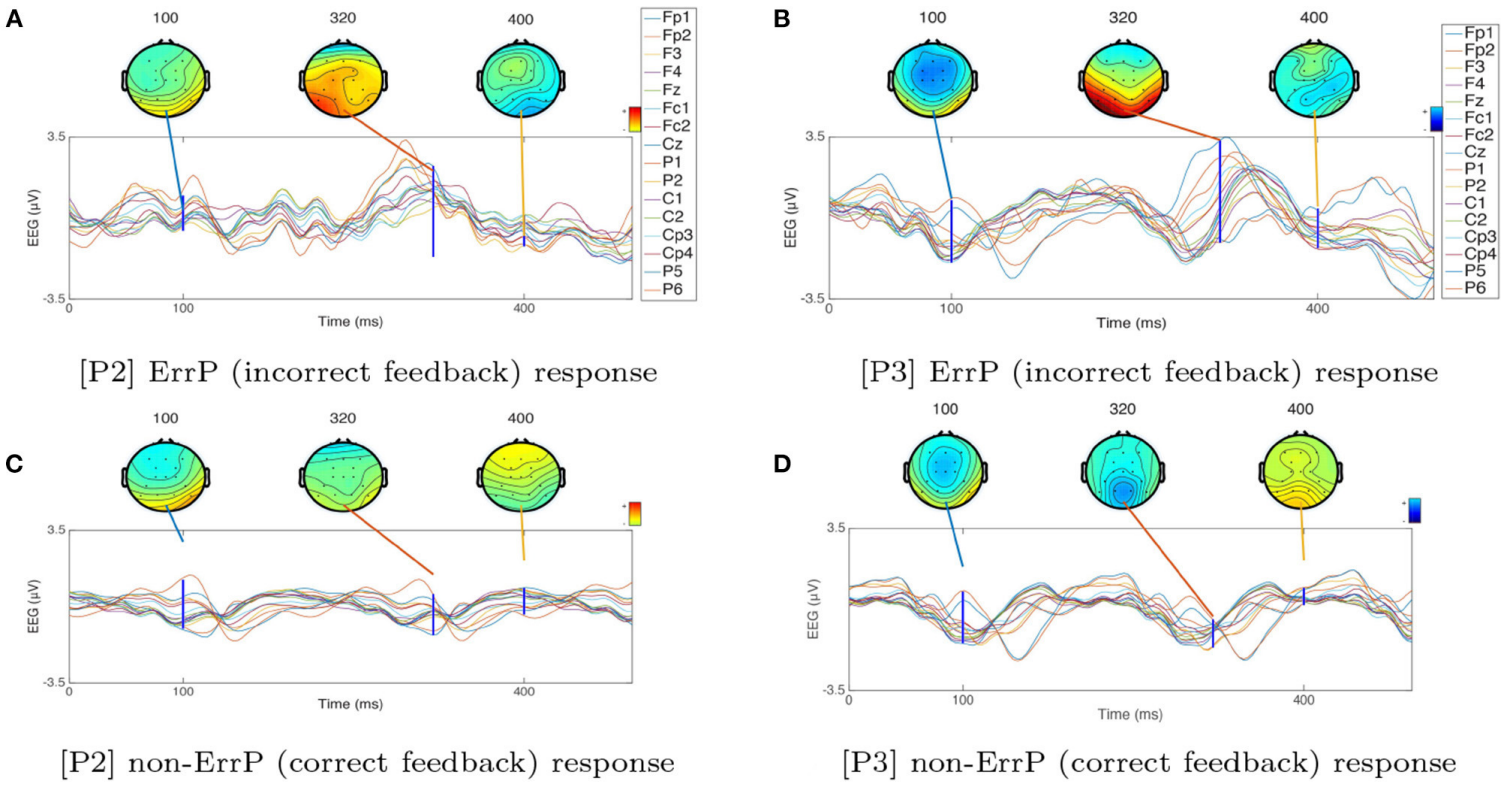
Average EEG responses of 12 users for **(A)** incorrect [P2], **(B)** incorrect [P3], **(C)** correct [P2] and **(D)** correct [P3].

**Figure 7:**
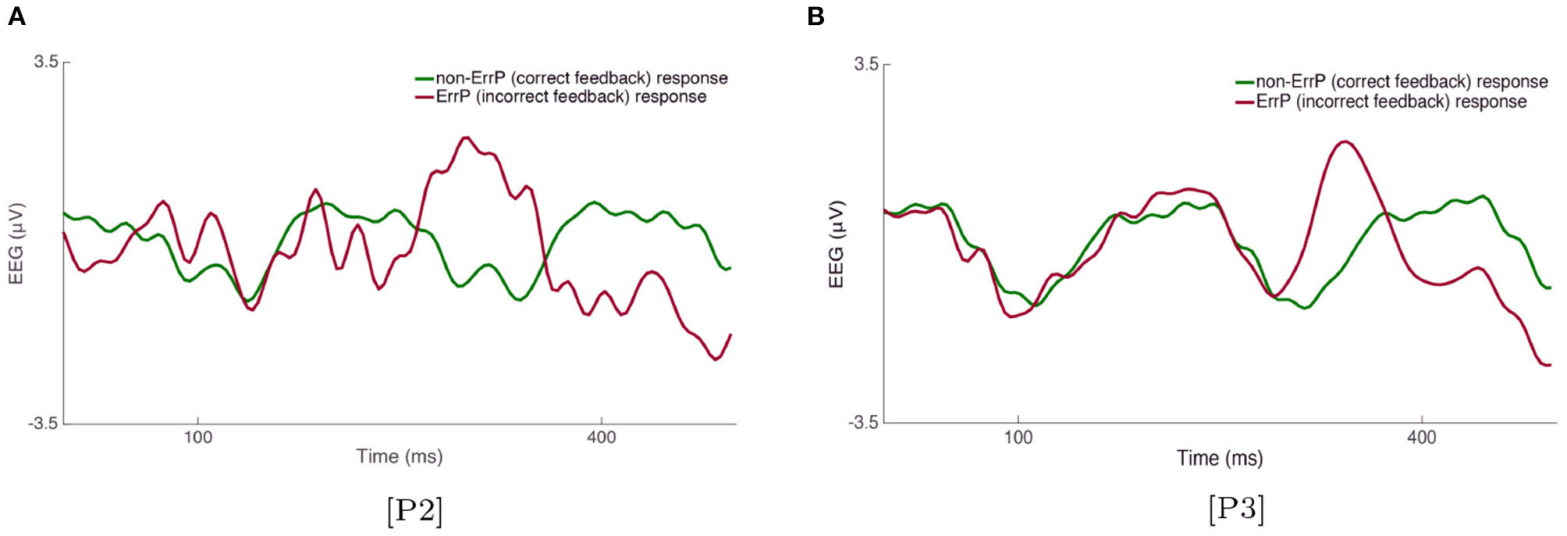
Average EEG response for correct and non-correct feedback of 12 users and 16 electrodes for **(A)** [P2] and **(B)** [P3].

Using the supervised data collected during the *calibration*_*FRP*_, we first analyze the average EEG recorded in response to correct and incorrect feedback for the two evidence acquisition, [P2] and [P3]. Figure 6 shows the average FRPs for the correct and incorrect feedback trials for 12 users for the two scenarios that use FRP; [P2] and [P3]. The results show the statistical presence of the ErrP response in both [P2] and [P3]. As we can see in Figures 6A,B, the waveform in response to the incorrect feedback is characterized by a positive component observed ([350 ms]) after the delivery of the incorrect feedback, representing a visually evoked potential (VEP). We do not observe this positive response after the correct feedback, as shown in Figures 6C,D. Upon the presentation of the incorrect feedback, a negative ([50–100] ms) component is also observed. In addition, Figures 6A–D show the scalp topography at different time windows {100, 320, 400} ms. Based on these results, we observe higher separability between the EEG time series recorded in response to non-correct and correct feedback around the time window of 320–350 ms. Finally, Figures 7A,B show the average FRPs for the correct and incorrect feedback for 12 users and 16 electrodes; for [P2] and [P3], respectively. From Figures 7A,B, we can observe that when [P3] paradigm is performed, the amplitude of this positive component ([350 ms]) for the incorrect stimuli is slightly lower compared to [P2] (it could be inferred from the amplitude difference between the green line (non-ErrP) vs. red line (ErrP) for [P2] and [P3]).)

**Figure 8:**
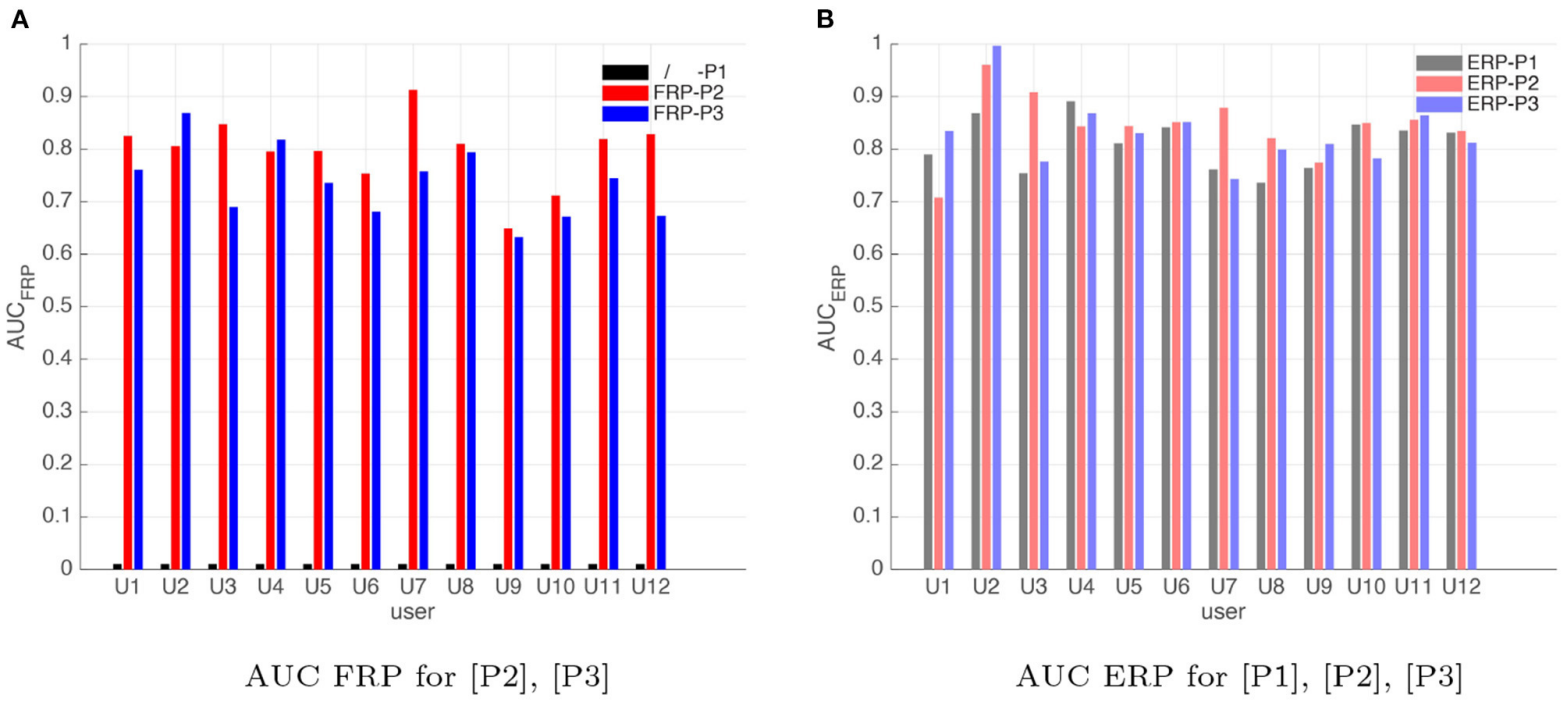
AUCs for 12 users for two calibrations tasks **(A)** *calibration*_*FRP*_, **(B)** *calibration*_*ERP*_, for 3 different evidence acquisition paradigms [P1], [P2], and [P3]. Please note that for [P1] we do not have calibration task FRP, since this paradigm does not use FRP evidence. The absence of FRP in [P1] is denoted with “/ - [P1]” and is shown in black. For [P1]: *AUC*_*FRP*_ = 0, *AUC*_*ERP*_ = 0.8138, for [P2]: *AUC*_*FRP*_ = 0.7966, *AUC*_*ERP*_ = 0.8308, for [P3]: *AUC*_*FRP*_ = 0.7341, *AUC*_*ERP*_ = 0.8300.

We then compare classification accuracies across different acquisition paradigms by employing AUC values as the measure of EEG evidence classification accuracy. In particular, using the calibration data obtained in the Human-in-the-loop calibration experiment (*calibration*_*ERP*_ and *calibration*_*FRP*_) as described in section 3.2, we compare the offline target vs. non-target stimuli and correct vs. incorrect feedback classification results for the three data acquisition paradigms, [P1], [P2], and [P3]. Figure 8A compares the areas under the receiver operating characteristics curves (AUCs) for the FRP evidences of each user in different acquisition paradigms, [P2] and [P3]. Similarly, Figure 8B shows the ERP classification AUCs for each user for different acquisition paradigms, [P1], [P2], and [P3]. AUC values are calculated based on the cross validation of the classifier's performance on the training (calibration) data sets. In 10 out of 12 users tested, the classification AUC for paradigm [P2] is larger than [P3], as observed in Figure 8A. This can be a result of the experiment [P2] being more controlled. In other words, since each RSVP sequence is appended with a prospect symbol in paradigm [P2], the user always knows when the feedback is going to be presented in [P2] as opposed to [P3]. Comparing the calibration results from Figures 8A,B we can see that for most users, the ERP calibration results have higher AUCs compared to the FRP classification. This difference in the classification AUCs can be due to the fact that the number of observations that we collect during ERP calibration is higher than the number of observations that we can collect during FRP calibration.

### 4.2. Human-in-the-Loop Copy Phrase Experiment Results

Using the data collected during three *copy phrase* tasks, we analyze the typing performance for the three evidence acquisition paradigms. As explained in section 3.2, each *copy phrase* task includes typing ten different phrases with different difficulty levels. Table 1 shows the typing accuracy performance of the three evidence acquisition paradigms for all users in terms of two measures: accuracy in typing a letter correctly (ATL), which is the total number of correctly typed letters divided by the total number of typed letters; and probability of the phrase completion (PPC) which is the total number of correctly typed phrases divided by the total number of phrases. We observe that both [P2] and [P3] paradigms improve the typing accuracy performance compared to [P1]. As shown in Table 1, none of the users are able to complete the 10 copy phrase tasks correctly using [P1]. A paired t-test is also performed on ATLs to compare the typing accuracies among different paradigms across 12 users. In most EEG-based BCI systems, signal recorded from multiple channels along the scalp is assumed to be a Gaussian process with an unknown covariance and mean (Gonzalez-Navarro et al., 2016b). Assuming the Gaussianity of the recorded signal, we believe that applying t-testing is plausible. The result is presented in **Table 3**. From **Table 3**, we observe very low p-values for [P2] vs. [P1], and [P3] vs. [P1]. However, no significant differences between [P2] and [P3] are observed.

**Table 1:** Typing performance results for 12 subjects performing a copy task using RSVP Keyboard™ for three different strategies [P1], [P2], and [P3].

	**P1**		**P2**		**P3**	
**User**	**ATL**	**PPC**	**ATL**	**PPC**	**ATL**	**PPC**
1	0.73	0.70	0.91	1.00	0.90	1.00
2	0.82	0.90	0.99	1.00	0.91	1.00
3	0.64	0.70	0.88	1.00	0.96	0.90
4	0.65	0.60	0.90	1.00	0.81	0.90
5	0.74	0.80	0.87	0.90	0.85	0.80
6	0.73	0.80	0.93	1.00	0.86	0.90
7	0.76	0.70	0.90	1.00	0.85	0.90
8	0.69	0.60	0.93	1.00	0.83	1.00
9	0.78	0.80	0.75	0.70	0.76	0.80
10	0.73	0.80	0.93	1.00	0.88	0.90
11	0.78	0.90	0.64	0.70	0.79	1.00
12	0.80	0.80	0.83	0.90	0.78	0.80
Mean	0.73	0.75	0.87	0.94	0.85	0.91

*ATL represents the accuracy in typing a letter correctly, and PPC is the probability of phrase completion*.

**Figure 9:**
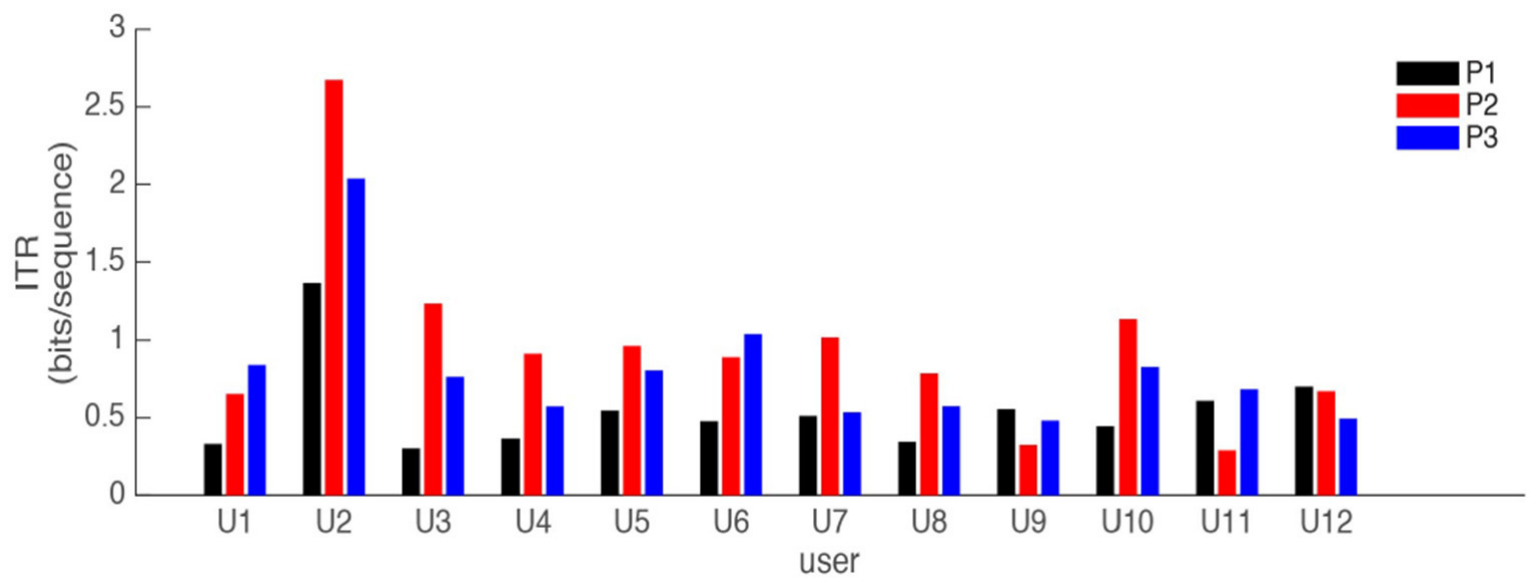
Average of information transfer rate (bits/sequence) for three evidence acquisition paradigms: [P1] (black), [P2] (red), and [P3] (blue). All 12 users performed the copy phrase task in RSVP Keyboard™.

Here, we use information transfer rate (ITR) (Obermaier et al., 2001) as another performance measure. ITR summarizes the accuracy and speed into a single metric and it is commonly used to measure BCI performance. Figure 9 illustrates the ITR (bits/sequence) values for all subjects; and Table 2 reports the mean of the ITR values among 12 subjects for the three strategies. From Figure 9 and Table 2 it can be observed that [P2] (in red) along with [P3] (in blue) yield considerable improvements in both speed and accuracy. Among all the results, [P1] displays the lowest performance. Using paired *t*-test, a hypothesis testing is also performed to compare the ITR values obtained from different paradigms across 12 participants. The results are presented in Table 3. Table 3 also represents [P2] as the slightly better paradigm, although the difference between [P2] and [P3] is not statistically significant.

**Table 2:** Mean of the ITR (bits/sequence) from Figure 9 for 12 subjects performing a copy task using RSVP Keyboard™ for three different strategies [P1], [P2], and [P3].

	**P1**	**P2**	**P3**
Mean ITR	0.55	0.96	0.80

**Table 3:** Hypothesis t-testing results for accuracy of typing a letter correctly (ATL) and ITR values for different evidence acquisition paradigms for 12 users.

**Paired *t*-Test Results between Different ATL Values**		**Paired *t*-Test Results Between Different ITR Values**	
***P*_*i*_ v.s. *P*_*j*_**	***P*-values**	***P*_*i*_ v.s. *P*_*j*_**	***P*-values**
[*P*1] v.s. [*P*2]	3.00*e*^−4^	[*P*1] v.s. [*P*2]	0.04
[*P*1] v.s. [*P*3]	9.20*e*^−5^	[*P*1] v.s. [*P*3]	0.06
[*P*3] v.s. [*P*2]	0.46	[*P*3] v.s. [*P*2]	0.47

*The null hypothesis is that the expected ATL/ITR difference of the two considered ITRs is zero. Here, we conducted a Bonferroni correction in which the critical significance level of α = 0.05 is adjusted*.

Human-in-the-loop copy phrase experiment results in Figure 9 and Table 1 show that the proposed strategies [P2] and [P3] outperform the strategy [P1] in terms of accuracy (with [P2] leading the race); and result in significant improvements in both speed and accuracy when compared to [P1]. We believe that improving not only accuracy but also speed is highly desired for BCI systems that are designed for real-life applications.

**Figure 10:**
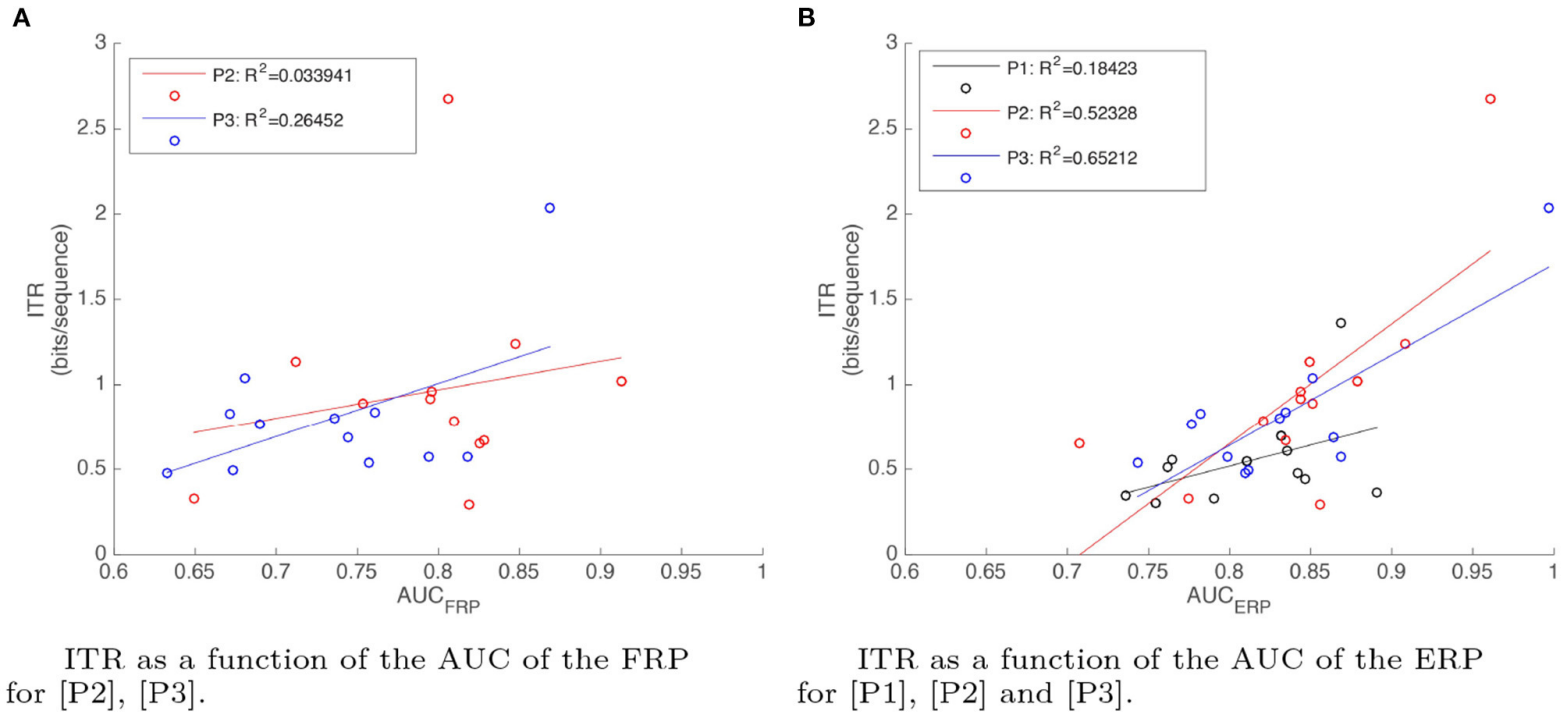
Linear regression relation between ITR (bits/sequence) and AUCs of: **(A)** FRP for evidence acquisition paradigms [P2] (red) and [P3] (blue), and **(B)** ERP for [P1] (black), [P2] (red), and [P3] (blue). Actual ITR values (represented by dots) and ITR values predicted by the linear model (represented by the solid line) are plotted as a function of the AUC for 12 users.

Finally, using online copy phrase and calibration results, we report ITR as a function of AUC obtained from the FRP and the ERP classifiers for each paradigm in Figures 10A,B. There are two different AUC values for paradigms [P2] and [P3] since they both use ERP and FRP evidences, whereas there is only one AUC value for [P1] corresponding to the ERP evidence. A linear regression model is fit to the observed data. We also report the coefficient of determination *R*^2^. From these figures it can be observed that in all three cases, there is a positive correlation between AUC and ITR. In the case of *AUC*_*ERP*_, the correlation is higher, which indicates that *AUC*_*ERP*_ is an effective factor to improve ITR for the three paradigms. For FRP, the correlation is much lower, so we can conclude that *AUC*_*FRP*_ has a small effect on ITR for both paradigms [P2] and [P3].

Compared to the benchmark BCI spellers which rely on visually evoked potentials (VEPs) such as SSVEPs, our ERP/ErrP based BCI speller has a slight advantage in accuracy (Liu et al., 2020), (Wong et al., 2020). Compared to the vision-independent BCI paradigms which rely on ERP elicitation via auditory and tactile stimulation, our visually evoked ERP/LM/FRP fusion BCI-speller has a significant advantage in ITR, and the accuracies we obtain with paradigms [P2] and [P3] compete with state-of-the-art P300 BCIs in the literature (Eidel and Kübler, 2020), (Kawala-Sterniuk et al., 2021).

## 5. Conclusions

In this manuscript, we compared three different Bayesian inference frameworks that tightly fuses context information and different EEG evidences to be used in intent inference engines of EEG-based brain computer interfaces. In particular, we study the potential benefits of fusing FRP, ERP, and language evidence using probabilistic generative models for a speller BCI. Based on the human-in-the-loop (copy phrase and calibration) experiments with 12 healthy participants using RSVP Keyboard™, three strategies are compared: [P1]-Baseline, which only fuses ERP/LM evidence; [P2]-AlwaysFRP, where each RSVP sequence is followed by an FRP trial using the top candidate in the alphabet according to posterior after ERP/LM evidence fusion; [P3]-ConfirmFRP, where the top candidate is shown as a prospect to generate FRP evidence only if its posterior exceeds a threshold, possibly after multiple ERP-evidence acquisition sequences.

We performed several analyses on the Human-in-the-loop copy phrase experiment results, which are: (i) accuracy (in the form of AUC, ATL, and PPC), (ii) speed (in the form of ITR), (iii) Information Transfer Rate (ITR) (bits/sequence). Our results show that by using enough FRP evidence in addition to ERP evidence and language model (LM), the typing speed could be increased compared to a model that does not use FRP evidence. Overall, both proposed strategies [P2] and [P3], which utilize FRP evidence outperform [P1] in terms of accuracy. Moreover, [P2] yields significant speed and accuracy and, therefore, ITR improvements compared to [P1] and also performs better compared to [P3]. These results could be due to the fact that for [P3] we do not collect enough FRP evidence during copy-phrase tasks, and that [P2] causes less mental fatigue due to its deterministic presentation method. We think that, for a Brain-Computer Interface which is designed to be used daily, it is crucial to improve the speed as well as the accuracy. Our results suggest that, probabilistic fusion of the FRP evidence can bring the true performance of a BCI one step closer to the objective.

According to the results, BCI users can benefit from the fusion of the FRP evidence to the decision making, if there are enough FRP evidences. Based on the analyses, we propose a BCI typing system capable of employing multiple evidence acquisition paradigms. This system, after individual assessments, will be able to determine the most profitable evidence presentation/inference paradigm as per user preference, capabilities, and EEG signal statistics.

We demonstrate theoretically that probing the users intent with FRP-acquisition using the current top candidate is an optimal strategy in an active learning framework employing the independent-trial-EEG-evidence assumption paradigm. This approach constitutes an improvement over previous literature employing ERP paradigms alone. In earlier work, we demonstrated that showing the top letters according to the current posterior in a sequence for ERP evidence acquisition is similarly optimal under the same independence assumption (Moghadamfalahi et al., 2015). Therefore, under the independent-trial-EEG-evidence model, the best strategy is to repeat the following until a decision is confidently made: *show the top candidate, gather EEG evidence, and update the posterior*. Clearly the independence assumption is incorrect, if not for the auto-correlation of EEG time series, due to the overlapping time windows that are used for trial-EEG-evidence extraction. Consequently, in an improved ERP/FRP/LM fusion framework that can be designed in the future, the following issues need to be considered more carefully: (1) a signal model that captures the temporal dependency of EEG features extracted for each trial, (2) the temporal cost of gathering a sequence-worth of ERP evidence vs. FRP evidence by showing the current top prospect. Therefore, in future work, we plan to address these issues and develop an ERP/FRP/LM fusion mechanism for BCI spellers that will dynamically decide whether to gather more ERP evidence, more FRP evidence, or neither during intent inference. The inference framework does not strictly rely on EEG evidence, therefore, we will also explore multi-modal physiological evidence fusion using signal sources such as EMG or eye-gaze trajectories.

## Data Availability Statement

The raw data supporting the conclusions of this article will be made available by the authors, without undue reservation.

## Ethics Statement

The studies involving human participants were reviewed and approved by Northeastern University Institutional Review Board. The patients/participants provided their written informed consent to participate in this study.

## Author Contributions

PG-N and DE conceived of the presented idea. PG-N and MM designed and performed the FRP experiments. All authors contributed to the analysis of the results and to the writing of the manuscript.

## Funding

This work was supported by NSF IIS-1149570, IIS-1118061, CNS-1136027, CNS-1544895, and SMA-0835976, by NIDR-H133E140026 and NIDLRR 90RE5017-02-01, and by NIH 5R01DC009834.

## Conflict of Interest

The authors declare that the research was conducted in the absence of any commercial or financial relationships that could be construed as a potential conflict of interest.

## Publisher's Note

All claims expressed in this article are solely those of the authors and do not necessarily represent those of their affiliated organizations, or those of the publisher, the editors and the reviewers. Any product that may be evaluated in this article, or claim that may be made by its manufacturer, is not guaranteed or endorsed by the publisher.
